# Scenario drafting to anticipate future developments in technology assessment

**DOI:** 10.1186/1756-0500-5-442

**Published:** 2012-08-16

**Authors:** Valesca P Retèl, Manuela A Joore, Sabine C Linn, Emiel JT Rutgers, Wim H van Harten

**Affiliations:** 1 Netherlands Cancer Institute-Antoni van Leeuwenhoek Hospital (NKI-AVL), Department of Psychosocial Research and Epidemiology, Plesmanlaan 121, Amsterdam, 1066 CX, The Netherlands; 2 Maastricht University, Department of Health, Organization, Policy and Economics, P.O. Box 616, Maastricht, 6200, The Netherlands; 3 Maastricht University Medical Center, Department of Clinical Epidemiology and Medical Technology Assessment, PO Box 5800, Maastricht, 6202 AZ, The Netherlands; 4 Netherlands Cancer Institute-Antoni van Leeuwenhoek Hospital (NKI-AVL), Department of Medical Oncology, Plesmanlaan 121, Amsterdam, 1066 CX, The Netherlands; 5 Netherlands Cancer Institute-Antoni van Leeuwenhoek Hospital (NKI-AVL), Department of Surgical Oncology, Plesmanlaan 121, Amsterdam, 1066 CX, The Netherlands; 6 University of Twente, School of Governance and Management, MB-HTSR, PO Box 217, Enschede, 7500 AE, The Netherlands

**Keywords:** Early technology assessment, Scenario drafting, Genomic profiling, 70-gene signature, Breast cancer

## Abstract

**Background:**

Health Technology Assessment (HTA) information, and in particular cost-effectiveness data is needed to guide decisions, preferably already in early stages of technological development. However, at that moment there is usually a high degree of uncertainty, because evidence is limited and different development paths are still possible. We developed a multi-parameter framework to assess dynamic aspects of a technology -still in development-, by means of scenario drafting to determine the effects, costs and cost-effectiveness of possible future diffusion patterns. Secondly, we explored the value of this method on the case of the clinical implementation of the 70-gene signature for breast cancer, a gene expression profile for selecting patients who will benefit most from chemotherapy.

**Methods:**

To incorporate process-uncertainty, ten possible scenarios regarding the introduction of the 70-gene signature were drafted with European experts. Out of 5 most likely scenarios, 3 drivers of diffusion (non-compliance, technical failure, and uptake) were quantitatively integrated in a decision-analytical model. For these scenarios, the cost-effectiveness of the 70-gene signature expressed in Incremental Cost-Effectiveness Ratios (ICERs) was compared to clinical guidelines, calculated from the past (2005) until the future (2020).

**Results:**

In 2005 the ICER was €1,9 million/quality-adjusted-life-year (QALY), meaning that the 70-gene signature was not yet cost-effective compared to the current clinical guideline. The ICER for the 70-gene signature improved over time with a range of €1,9 million to €26,145 in 2010 and €1,9 million to €11,123/QALY in 2020 depending on the separate scenario used. From 2010, the 70-gene signature should be cost-effective, based on the combined scenario. The uptake-scenario had strongest influence on the cost-effectiveness.

**Conclusions:**

When optimal diffusion of a technology is sought, incorporating process-uncertainty by means of scenario drafting into a decision model may reveal unanticipated developments and can demonstrate a range of possible cost-effectiveness outcomes. The effect of scenarios give additional information on the speed with cost effectiveness might be reached and thus provide a more realistic picture for policy makers, opinion leaders and manufacturers.

## Background

Especially in early stages of promising new technologies, Health Technology Assessment (HTA) information should be used to anticipate possible developments. Performing a HTA requires sufficient patient numbers and, as a consequence, broad clinical implementation of new technologies may be premature in the absence of firm prospective data on the actual benefits [1]. However, if we wait to perform a HTA, it might very well be that worthwhile technology is withheld from the public [2]. This paradox has become known as Buxton’s law: *“It is always too early, until suddenly, it is too late…”*[3]. We feel that there is a need to integrate methods in TA for dealing with the various possible developments in early stages of technology development, both to support information for policy makers, opinion leaders and manufacturers, and to anticipate developments encountered during the early introduction in clinical practice. Combining structured scenario drafting and decision modelling could be helpful to integrate these dynamics when calculating expected effects and costs.

The question is whether HTA –in the broad sense of the term- can be conducted in advance of widespread adoption of a technology? This question has also been featured by a rich body of publications in the recent years [4-7], however, none of these articles focused on incorporation of qualitative scenarios from the perspective of various stakeholders into a cost-effectiveness model.

An example of a promising technique in its early stages of development is the 70-gene prognosis signature (MammaPrint^TM^) for breast cancer patients [8]. Using the 70-gene signature, the selection of patients that will benefit most from chemotherapy could be more accurate compared to currently used clinical guidelines, and thereby reducing over-treatment. The promising results of three retrospective validation studies [9-11] led to the performance of a prospective feasibility study (RASTER: Microar**RA**y Progno**ST**ics in Breast Canc**ER**) from 2004 until 2006 [12], followed by a prospective, randomized clinical trial (MINDACT: Microarray In Node-negative Disease may Avoid ChemoTherapy), from 2007 until 2011 [13].

It would take at least 8–10 years to bring the signature into routine clinical practice via the usual path of prospective trials [14]. It was therefore decided that the controlled introduction of this technology, starting in 2004, should be supported by an early and dynamic form of HTA; Constructive Technology Assessment (CTA).

CTA is based on the idea that during the course of technology development, choices are constantly being made about the form, the function, and the use of that technology [15,16], and attempts to influence the development and diffusion of a new technology in a beneficial way [17].

For the introduction of the 70-gene signature, the CTA-part focused on quality aspects that were most likely to change during the introduction, such as: efficiency, logistics, ethical/legal aspects, patient centeredness and cost-effectiveness [18-20]. From the cost-effectiveness analysis was learned that the 70-gene had the highest probability of being cost-effective compared to the currently used clinical guidelines St. Gallen guidelines [21] and the Adjuvant! Online software [22] for a willingness to pay more than €4,614/QALY [20].

Simultaneous with the early introduction, scenarios were drafted to monitor and anticipate these changing aspects, in other words: the dynamics of the 70-gene signature diffusion. The scenarios were written in two steps; firstly, two overall scenarios were developed and secondly, a scenario workshop with more detailed scenarios was organized.

The technology-related developments and the diffusion pathway of the 70-gene signature are likely to have impact on the cost, effects and cost-effectiveness in the future. In cost-effectiveness analyses (CEAs), it is common to use different quantitative scenarios in sensitivity analyses to reflect the uncertainty of input-parameters [23]. There are only a few examples in the literature where more comprehensive, qualitative scenarios were processed into a CEA [24,25].

Our research objectives were: first, to develop a multi-parameter framework to assess dynamic aspects to determine the effects, costs and cost-effectiveness of possible future diffusion patterns of technologies at an early stage of development. Second, to illustrate this method for the 70-gene signature versus the current Adjuvant! Online (AO) treatment strategy for breast cancer patients.

## Methods

The following steps in the multi-parameter framework can be distinguished: (I) determination of the phase of diffusion; (II) scenario construction; (III) grouping of scenarios; (IV) integration of the factors as parameters in the decision model; (V) input model parameters; (VI) model analysis.

### Determination of the phase of diffusion

**Figure 1:**
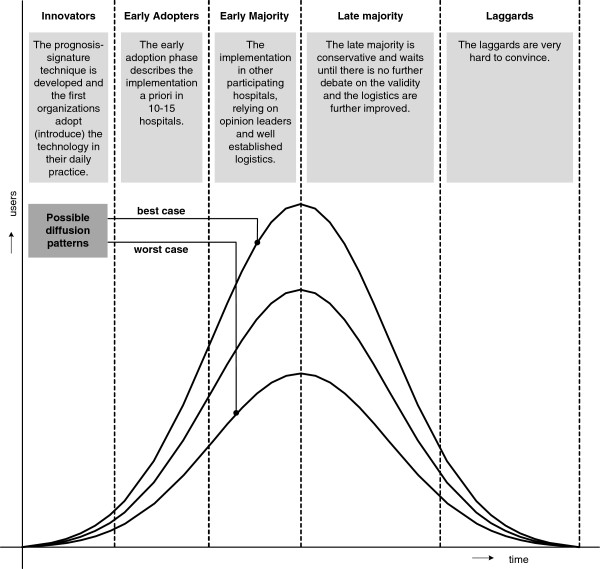
Rogers’ adoption curve with possible diffusion patterns.

To position the scenarios in a timeline, we used the ‘diffusion theory’ of Rogers [26], where several phases reflect the diffusion path of the technology related to the numbers of adopters (Figure 1). “Diffusion is the process by which an innovation is communicated through certain channels over time among the members of a social system” [26]. The possible developments as described in a scenario can have influence on the degree and speed of diffusion of the technology in clinical practice.

In the case of the 70-gene signature, the diffusion pathway is described following the curve as in Figure 1: *innovation phase (2003–2005)*, the prognosis signature technique was developed and the first organisations (innovators) adopted the technology in their daily practice. The *early adoption phase (2005–2007)* describes the implementation in 10–15 hospitals: the logistics were established and physicians increasingly based their adjuvant treatment decision on the signature result. The *early majority phase (2007–2012 and beyond)* describes the implementation in a gradually increasingly number of hospitals participating in the prospective randomised controlled MINDACT-trial [27].

### Scenario construction

The method we used to construct the scenarios was based on the Shell (Royal Dutch Oil multinational) approach, using a most likely course of development with ‘There Is No Alternative’ (TINA) elements and the dynamic nature introduced by ‘what if’-deviations [28]. The Shell method consists of background research, drafting one or two scenarios, structured feedback by experts and revision of these drafts [29,30]. In fact, the scenario planning concerns planning based on the systematic examination of the future by picturing plausible and consistent images thereof. A specific element in this method is the introduction of “what if” deviations; especially in early stages the degree of uncertainty may render it helpful to consider alternatives. The Delphi method, in turn, attempts to develop systematically expert opinion consensus concerning specific aspects of future developments and events. It is a judgmental decision and/or forecasting procedure in form of an anonymous, written, multi-stage survey process, where feedback of group opinion is provided after each round. Generally speaking, the output of the different phases of the Delphi method can be used as input for the scenario method. More background details will be found at the Shell website [28] and a former publication concerning this subject [15].

We used a semi-structured questionnaire to present ten scenario related options as “What if…” statements to genomic experts and breast cancer specialists; members of the TRANS- breast international group (TRANSBIG). These are the health care professionals that are going to use or work with the 70-gene array in the (future) daily practice. The responses of the questionnaires were used as feedback for the ultimate scenarios presented at a workshop with a formal decision procedure, which was attended by 80 participants (surgeons, medical oncologists, molecular pathologists and radiotherapists). These experts were asked to vote on the 10 scenario options; they had to indicate, whether each alternative was “likely” or “unlikely” to happen within 10 years. The resulting scenarios are described in Table 1.

**Table 1:** Scenario results and likelihoods derived from the workshop with experts

**Workshop Scenario**		**Description**
1	Hesitant adopters (100% likely)	Professionals, who are not using the 70-gene signature until the results of the MINDACT are released, will delay the diffusion (spreading of the signature) process. This will be expressed in the proportion of non-compliance towards the signature result.
2	User-friendliness (90% likely)	There is a mix of new functions possible on the (read-out) microarray; such as ER/PgR/Her2 status, singles genes, with new possibilities for e.g. targeted therapies. Furthermore, by using needle biopsies the application becomes more user-friendly. This will be expressed in a decrease of failures of the signature.
3	Progressive techniques (90% likely)	There is positive proof for the value of RNA-preservation instead of formalin-based tissue for future research, which causes an increased use of the 70-gene signature. This will be expressed in a decrease of failures of the signature.
4	Progressive uptake (90% likely)	The 70-gene signature has developed further and can be used safely for all node negative and 1–3 positive patients. The uptake is 100% in your county and is embedded in the national guidelines. This will be expressed in an increasing number of patients receiving signature.
5	Financial access (75% likely)	The insurance companies in the Netherlands don’t reimburse the use of the 70-gene signature yet (2008). If the insurers were to reimburse the 70-gene signature, the rate of reimbursement agreements would be rather more progressive throughout Europe. This will be expressed in a –slightly slow- increase of patients receiving the signature.
6	Other paraffin/test (60% likely)	Another PRC-based, user-friendly test appears on the market, and the market share of the 70-gene signature decreases.
7	Competitive test (60% likely)	The Oncotype DX ‘wins’ the competition; the market share of the 70-gene signature decreases.
8	Era after: CTC? (40% likely)	A totally new (nano) technology has been developed (using fresh frozen tumour samples) which has more value than the 70-gene signature and - due to this test - the market share of the 70-gene signature decreases.
9	Provision on free market (18% likely)	Besides being used in the MINDACT trial, 70-gene signature is also available on the free market, to prevent unethical situations due to patient selection.
10	Regulation/legislation barrier (5% likely)	There is a probability of legal regulation by way of FDA clearance. Because the 70-gene signature has FDA and IVDMIA (In Vitro Diagnostic Multivariate Index Assay) approval, the market share of the Oncotype DX decreases.

CTC: circulating tumour cells, ER/PgR: oestrogen and progesterone receptors.

### Grouping of scenarios

**Figure 2:**
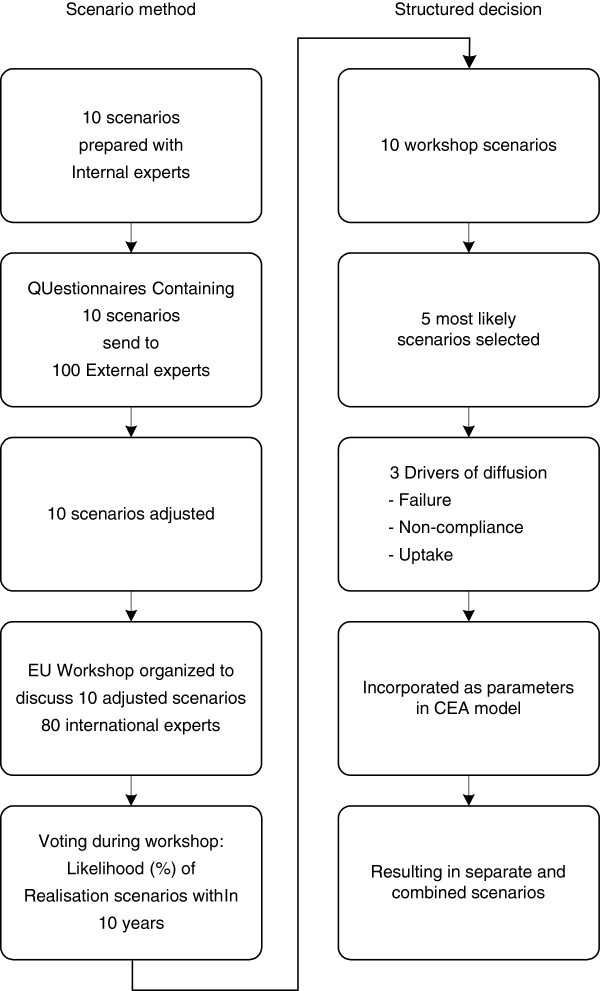
**Scenario method and structured decision, based on the Shell method**[28].]

For the scenarios to be incorporated in the cost-effectiveness modelling we used a structured decision process (Figure 2). From the ten “workshop” scenarios, the five most likely were selected by ranking the likeliness of the scenarios. The most crucial accelerating or decelerating aspects (drivers of the diffusion) were identified which resulted in three main factors: technical failure, non-compliance with discordant test results, and uptake. Technical failure was based on the “user-friendliness” and “RNA preservation” workshop-scenario. Non-compliance was based on the “hesitant adopters” scenario. We noticed that from the moment the first papers appeared quite some opinion leaders were rather sceptical to the value of the test, which related mostly to methodological issues and possibly to the lack of knowledge on this new genomic and medical technical domain. As this seemed to defer the stages of diffusion after innovation and early adoption (cf Rogers) and strong criticisms were voiced, we labelled these “hesitant adopters”. Uptake was based on the “financial access” (moderate increase in uptake), and the “progressive uptake” scenario (rapid increase in uptake). The three drivers of diffusion were incorporated as parameters in the decision model, see Table 2.

**Table 2:** Input parameters

**Workshop scenario**		**Barrier or facilitator?**	**Drivers of diffusion (Parameter in decision model)**	**Mean parameter value in decision model**		**Source**
1	Hesitant adopters	Barrier	Non-compliance	2005	0.35	12
				2010	0.26	Scenario workshop
				2020	0.08	Scenario workshop
2 & 3	User friendliness & Progressive techniques	Barrier	Technical failure	2005	0.27	12
				2010	0.20	Scenario workshop
				2020	0.08	Scenario workshop
4 & 5	Progressive uptake & Financial access	Facilitator	Uptake	2005	0.03	12
				2010	0.50	Scenario workshop
				2020	0.92	Scenario workshop

### Integration of the driving factors as parameters in the decision model

**Figure 3:**
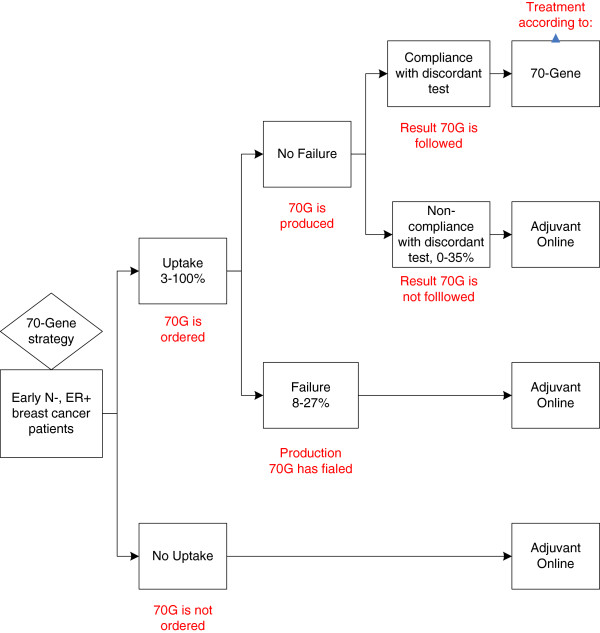
Scenario parameters as calculated in the model.

A Markov decision model was previously developed to assess the effects (quality-adjusted life years; QALYs), costs and cost-effectiveness of the 70-gene signature compared to clinical-pathological guidelines (such as Adjuvant! Online [22]) for patients aged 50 years with early, operable, node-negative, oestrogen receptor (ER) positive breast cancer. In each strategy, based on the sensitivity and specificity of the prognostic test calculated from a pooled analysis consisting of 3 previously reported validation studies, patients were classified as having a true low, true high, false low, or false high risk of developing metastasis. It was assumed that both the prognostic test result and the treatment guidelines would be followed in all cases. We simulated in the model that all patients received endocrine treatment, and in case of a high risk, the patient received also chemotherapy. The model was constructed with four mutually exclusive health states: disease free survival, relapse (including local and regional recurrences, secondary primary and contralateral breast cancer), distant metastasis, and death (Figure 3). It was assumed that patients could only have one relapse, for which they received the best available treatment with the same costs, regardless which kind of adjuvant treatment the patient originally received for the primary tumour. The calculations were performed per year, with a total simulated time horizon of 20 years. The analyses were performed from a health care perspective from the Netherlands. Future costs and effects were discounted to their present value by a rate of 4% and 1.5% per year respectively, according to Dutch guidelines [31]. Costs were expressed in 2005 Euros. We programmed the model in Microsoft Excel (Microsoft, Redmond, WA).

**Figure 4:**
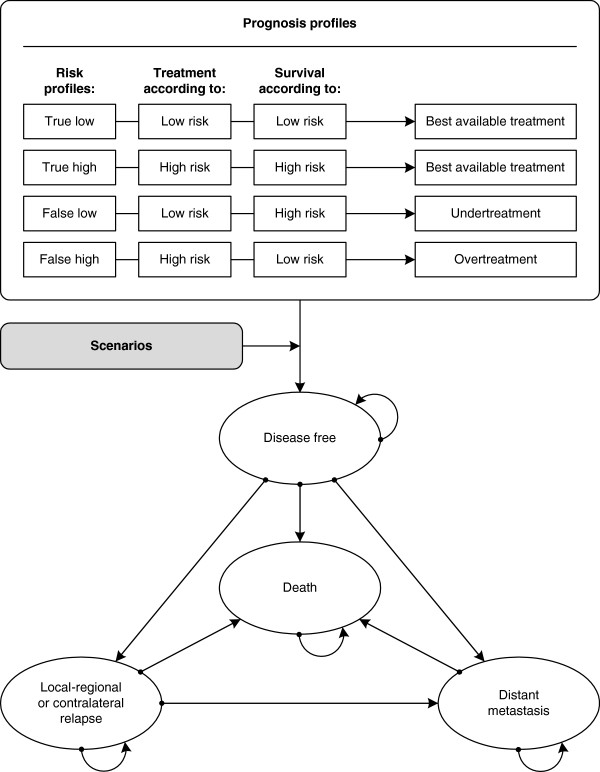
Model structure.

In case of a technical failure, mainly due to insufficient tissue quantity or -quality, the 70-gene signature could not be performed and no result could be delivered to the physician and patient. In some of these cases preparatory costs were already made. It was assumed that in these cases 10% of the total costs of the 70-gene signature were made and the final treatment advice was according to the clinical guideline (AO). In case of non-compliance with a discordant test result, low risk signature and high risk AO or vice versa, the 70-gene signature result was available, but not used in the adjuvant treatment decision. It was assumed that patients would thus be treated according to the Adjuvant! Online result. The uptake parameter reflected the proportion of the target population (patients who actually did receive the 70-gene signature divided by all patients who are in principle eligible for the signature (target population), either 50% in 2010, in case of the “financial access” scenario, and 92% in 2020, in case of the “progressive uptake” scenario (Figure 4).

### Input parameters

To reflect the dynamics in the diffusion, values of the parameters were changed over time. Cost-effectiveness was assessed for three points in time: 2005 (early adoption, data available), 2010 (early majority phase, data based on scenarios), 2020 (late majority, data based on scenarios). All scenarios starting in 2005 were based on data from the RASTER study [12], as well as the uncertainty, which was assumed to stay constant over time. The initial value of the technical failure parameter was 27%, as this occurred in the total available samples in the RASTER-study. Based on the workshop results, we assumed that the 27% failure rate would be reduced to 20% in 2010 and to 14% in 2020. Non-compliance was modelled in case of a clinical high/genomic low risk (15% in the RASTER-study) and in case of a clinical low/genomic high risk (20%); thus in total 35% non-compliance. Based on the scenarios, the total non-compliance was likely to reduce to 26% in 2010 and to 8% in 2020, assuming a positive result of the MINDACT-trial. The “uptake” parameter was calculated with the numbers of patients who annually received a 70-gene signature divided by the incidence of the targeted group in the Netherlands. We used the numbers of signatures performed in the RASTER-study (n = 427) to feed the data of 2005 [12]. This specific parameter could be negatively influenced by the “financial access” scenario, where the uptake of the 70-gene signature is delayed by insurance companies who do not reimburse the signature; or a competitor test could enter the market with serious effects on the likely sales, which we modelled with an uptake probability of up to 50% in 2010. The uptake parameter could subsequently be positively influenced by the “progressive uptake” scenario, where the 70-gene signature would be adopted optimally in Europe and embedded in guidelines in up to 92% of cases in 2020.

### Model analysis

The three “drivers of diffusion” parameters; failure, non-compliance and uptake were separately integrated in the Markov model, by changing only one specific parameter and leaving the others fixed. In addition, the parameters were combined per year; a 2010 scenario (combination of the failure, non-compliance and adoption scenarios in 2010) and a 2020 scenario (combination of the failure, non-compliance and adoption scenarios in 2020). For each scenario, the incremental costs, effects and incremental cost-effectiveness ratio (ICER) of the 70-gene signature versus the Adjuvant! Online were calculated for 2005, 2010 and 2020. Incremental effects and incremental costs were obtained by subtracting the effects or costs of the Adjuvant! Online strategy from the 70-gene signature strategy. The ICER was calculated by dividing the difference in expected costs (Δ*C*) by the difference in expected effects (ΔE) compared to a certain threshold value (λ) [32].

(1) iNMB=ΔE*λ−ΔC

An ICER lower than the threshold implies that the 70-gene signature is cost-effective compared to the AO, and an ICER higher than the threshold implies that the 70-gene signature is not cost-effective. The threshold reflects the maximum willingness to pay of the society, whether a strategy is deemed efficient depends on how much society is willing to pay for a gain in effect, which is referred to as the ceiling ratio [32]. As a threshold for a positive decision on coverage, we used €30,000 per QALY, which reflects the £20,000-30,000 per QALY applied by the National Institute for Health and Clinical Excellence (NICE) [33]. Parameter values were drawn at random from the assigned distributions, using Monte Carlo simulation with 1000 iterations. Uncertainty in the input parameters was handled probabilistically, by assigning distributions to parameters. To show decision uncertainty, cost-effectiveness acceptability curves (CEACs) frontiers are presented [34].

## Results

### Mean results

For the start in 2005, the effects and costs for the 70-gene compared to the Adjuvant! Online strategy were almost equal; the incremental (difference in) QALYs were 0.0010 and the incremental costs amounted to €1,940 (Table 3). Over time, improvement was shown per separate scenario. The technical failure scenario resulted in incremental effects of 0.0011 (2010) and 0.0013 (2020), and incremental costs of €2,094 in 2010; and €2,385 in 2020. The observed higher costs for the 70-gene, were due to more successful tests. The reduction of non-compliance showed incremental effects of 0.0011 (2010) and 0.0013 (2020), and incremental costs of €1,939 in 2010 and €1,940 in 2020. The uptake scenario resulted in incremental effects of 0.0592 in 2010 and 0.1089 in 2020, and incremental costs of €1,547 in 2010, and €1,211 in 2020.

**Table 3:** Mean results, incremental effects, costs, cost-effectiveness ratio and Incremental Cost-Effectiveness Ratio (ICER)

**Model scenario**	**time**	**Mean value parameter**			Δeffects*	Δcosts*	**ICER**
		**failure**	**NC**	**uptake**			
Start	2005	0.27	0.35	0.03	0.0010	€ 1,940	€ 1,9 mill
***Separate scenarios***							
Failure	2010	0.20	Idem	Idem	0.0011	€ 2,094	€ 1,9 mill
	2020	0.08	Idem	Idem	0.0013	€ 2,385	€ 1,9 mill
Non-compliance	2010	Idem	0.26	Idem	0.0011	€ 1,939	€ 1,7 mill
	2020	Idem	0.08	Idem	0.0013	€ 1,940	€ 1,5 mill
Uptake	2010	Idem	Idem	0.50	0.0592	€ 1,547	€ 26,145
	2020	Idem	Idem	0.92	0.1089	€ 1,211	€ 11,123
***Combined scenarios***							
2005		0.27	0.35	0.03	0.0010	€ 1,940	€ 1,9 mill
2010		0.20	0.26	0.50	0.0728	€ 1,630	€ 22,388
2020		0.08	0.08	0.92	0.1492	€ 1,171	€ 7,853

Δeffects: Incremental effects of 70-gene signature compared to the Adjuvant Online, Δcosts: Incremental costs of 70-gene signature compared to the Adjuvant Online, ICER: Incremental cost-effectiveness ratio, NC: non compliance.

**Figure 5:**
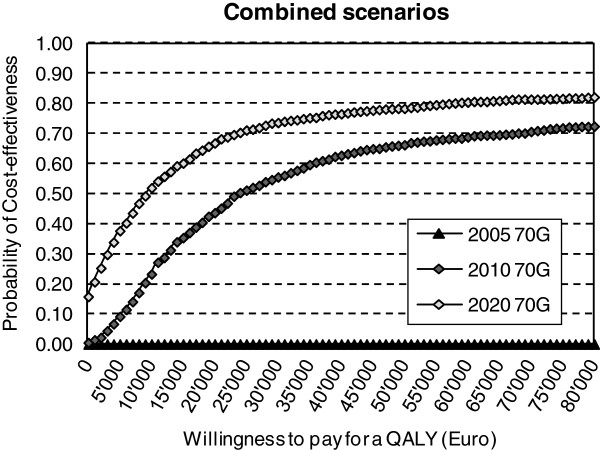
Cost-Effectiveness Acceptability Frontiers for the combined “drivers for diffusion” scenarios.

Assuming a maximum willingness to pay of €30,000/QALY, in 2005 the 70-gene signature was not yet cost-effective compared to the current clinical guideline, with an ICER of €1,9 million/quality-adjusted-life-year (QALY). The ICER for the 70-gene signature improved over time with a range of €1,9 million to €26,145 in 2010 and €1,9 million to €11,123/QALY in 2020 depending on the separate scenario used (Figure 5). The combined scenarios of 2005, 2010 and 2020 showed that the probability of cost-effectiveness of the 70-gene signature for a maximum willingness to pay of €30,000/QALY is 56% in 2010, and will be 73% in 2020. In general, the uptake scenarios generated the greatest impact on cost-effectiveness, followed by technical failures and non-compliance.

## Discussion

In the case of the 70-gene signature, the results of the scenarios incorporated in the dynamic CEA showed a wide range of possible ICERs over time. The effect of uptake, failure and non-compliance give additional information on the speed with cost effectiveness might be reached and thus provide a more realistic picture for policy makers. In our view it might more often be the case that real world variations are not accounted for in cost-effectiveness analyses, as these often rely on information from randomized controlled trials [35]. Besides for policy makers, this information can also be valuable to guide implementation efforts and/or product improvements.

This article shows that, in the absence of sufficient data, scenarios can help to anticipate the future diffusion patterns and use of technology by providing insight into future developments. When integrated in a decision model, these scenarios can also improve the ability to make an informed policy decision. An advantage is that scenario-discussion and -analysis reveals factors that can be anticipated and may warrant intervention in the implementation process, in order to stimulate “appropriate use” and optimal cost-effectiveness at a population level.

In the case of the 70-gene signature, the influence of the uptake scenario seemed to generate the highest impact on the cost-effectiveness results. As the uptake of the 70-gene signature increases, the net benefit will obviously increase and the 70-gene becomes cost-effective. Informing doctors and patients and generating additional evidence, for instance through “coverage with evidence development” program, are possible means to enhance uptake. When comparing the improved compliance results with the reduction of failure results, failure seemed to generate larger impact on cost-effectiveness, mainly due to remaining costs for tests which failed throughout the process. Compliance improvement was observed actually in the pilot study of the MINDACT with a total of 5 % non-compliance in the discordant cases [27]. This was, however, measured in a trial design, which may not be representative for use of the 70-gene signature. Hesitant adopters will have the confidence after prospective data is been released and ease of use could be established by using the 70-gene signature in decision making integrated into the Adjuvant! Online software as a hazard rate.

There are some remaining issues with regard to the scenario method used. First, to keep the analysis stable, we modelled the uncertainty constant over time. It is true expecting that the uncertainty will decrease in the future, but for the ease of comprehension, we left this stable. We expect uncertainty will decrease over time, for example when the results of the MINDACT trial will become available. This is likely to make the results of our analysis more favourable for the 70-gene signature. By using value of information analysis (VOI) one can characterise, and possibly deal, with uncertainty. We are currently exploring these approaches [36,37]. Second, the uptake scenario turned out to be most influential, however, we could only use the numbers of the studies conducted in 2005 and 2010, in real there could be a lot more signatures used, and thereby effect the cost-effectiveness of the 70-gene in a positive way. Furthermore, the reimbursement decision in this case is based on knowledge and ideas regarding the accuracy of the 70-gene signature. This will indeed either accelerate or slow down the uptake of the signature. In the scenarios, we took into account a (delayed) uptake of 50% in 2010, which is in fact the threshold for cost-effectiveness of the 70-gene signature. Thus in case the uptake will stay below the 50%, the 70-gene signature will not be considered cost-effective. Thirdly, in the scenario workshop, most of the participants were already (short or long) working with the 70-gene signature in clinical practice. In our opinion, the experts who participated in the workshop had a clear view of the potential barriers and facilitators, because of their large experience and because they originated from different EU countries. However, more participants in the late majority or laggard phases could possibly have changed the scenario likelihoods. As a result, the scenarios might be more of a decelerating character, which would lead to higher costs and less effect for the 70-gene signature. In addition, alternative methods are available to elicit likelihoods of different scenarios, such as a Delphi-like approach. Finally, it is possible that costs of drugs used in adjuvant chemotherapy regimens may be underestimated because the costs of Taxanes, used in adjuvant chemotherapy regimen, are expected to increase in the coming years [38].

The discussed method made it possible to integrate qualitative scenarios into quantitative parameters and derive scores from experts in order to obtain an impression on the most likely future developments. A next phase could be to derive more quantitative scenarios, by preparing the choices for the experts in a more quantitative way, as has been described by some authors [39], and by evaluating the different options against each other. Another point of further research could be the exact timing of performing a CTA using scenarios and (retrospective) confirmation in other studies that dynamic CTA is possible.

With respect to the 70-gene signature, there is likely to be more than one “truth” regarding cost-effectiveness, especially in early stages of development. If we consider expected costs and outcomes, we cannot be certain about future developments. Technology Assessments in uncertain diffusion phases may be occurring more often; especially early stage cancer where researchers have to wait up to 10–20 years for relevant outcome data. Current advances in understanding cancer biology have provided leads to develop new, effective targeted therapies. However, progress is slowed by suboptimal/outdated clinical trial design paradigms and by regulatory complexity and rigidity. Ongoing studies like the Investigation of Serial studies to Predict Your Therapeutic Response with Imaging And MoLecular analysis (the ISPY-trial) are recent examples that using a new endpoint in the analyses (pathological complete response (PCR)) can be considered to evaluate study results at an earlier stage [40].

It is important to support those studies with a CTA in order to monitor developments and anticipate them at an early stage. Structured scenario drafting can be used as a tool in this process, and seems especially suited to integrate in decision-analytical models. This ultimately provides the decision maker, opinion leader and/or manufacturer with early, more detailed information of possible developments and of a likely range of cost-effectiveness results of a clinical technology, and the aspects that can be relevant, to improve the product or guide further diffusion.

## Abbreviations

(H)TA, Health Technology Assessment; RASTER, MicroarRAy PrognoSTics in Breast CancER; MINDACT, Microarray In Node-negative Disease may Avoid ChemoTherapy; CTA, Constructive Technology Assessment; AO, Adjuvant Online software; CEA, Cost-Effectiveness Analysis; TRANSBIG, TRANS-Breast International Group; RNA, Ribonucleic acid; ER, Estrogen receptor; NICE, National Institute for Health and Clinical Excellence; QALY, Quality Adjusted Life Years; ICER, Incremental Cost Effectiveness Ratio; CEACs, Cost-effectiveness acceptability curves; CI, Confidence Interval; ISPY, Investigation of Serial studies to Predict Your Therapeutic Response with Imaging And MoLecular analysis; PCR, Pathological Complete Response; NC, Non-compliance; UP, Uptake; SW, Scenario Workshop.

## Competing interest

Prof. Dr. W.H. van Harten is a non-remunerated, non-stake holding member of the supervisory board of Agendia Inc. All other authors declared no conflicts of interest.

## Authors’ contributions

VR & MJ performed the cost-effectiveness analysis, VR, WH & ER carried out the acquisition of the (scenario) data, VR & WH drafted the manuscript. MJ has made substantial contributions to the conception and design to the study and co-drafted the manuscript. SL carried out the acquisition of the (scenario) data and has made substantial contributions to the conception and design to the study and revised the manuscript critically. WvH, SL & ER participated in its conception and design and coordination and helped to draft the manuscript. All authors read and approved the final manuscript.

## Source of funding

This study was funded by the Dutch Health Care Insurance Board (DHCIB), the Netherlands. (M03ARR): “mRNA expression patterns as an aid in adjuvant systemic therapy decision making in lymph node negative breast cancer patients.” (RASTER-trial) The DHCIB had no role in the study design or in data collection, analysis and interpretation, and it was not involved in the decision to publish.
